# Supplementary material for: Diversity and Functional Distribution Characteristics of Myxobacterial Communities in the Rhizosphere of Tamarix chinensis Lour in Ebinur Lake Wetland, China
Source: Microorganisms. 2023 Jul 28;11(8):1924. doi: 10.3390/microorganisms11081924 (PMC10459050; doi:10.3390/microorganisms11081924)
Supplement: Supplementary file 1 [file microorganisms-11-01924-s001.zip › supplementary files/Table S1.pdf]

Supplementary

Supplementary Figures and Tables

Table S1.

Table S1 PERMANOVA analysis of genus horizontal myxobacteria communities in three seasons.

pairs	Df	Sums of squares	F.Model	Variation (R2)	p.value	p.adjusted
Group	2	0.346007203	0.173003601	2.45270571	0.153748571	0.026
cL4 vs cL7	1	0.067108601	1.239304716	0.064415255	0.252	0.252
cL4 vs cL10	1	0.273595534	3.104002747	0.147081233	0.028	0.084
cL7 vs cL10	1	0.178306669	2.572431939	0.125042676	0.058	0.087
