# Supplementary material for: Diversity and Functional Distribution Characteristics of Myxobacterial Communities in the Rhizosphere of Tamarix chinensis Lour in Ebinur Lake Wetland, China
Source: Microorganisms. 2023 Jul 28;11(8):1924. doi: 10.3390/microorganisms11081924 (PMC10459050; doi:10.3390/microorganisms11081924)
Supplement: Supplementary file 1 [file microorganisms-11-01924-s001.zip › supplementary files/Table S2.pdf]

Table S2

Table S2 PERMANOVA analysis of genus-level myxobacteria communities in different spaces.

pairs	Df	SumsOfSqs	F.Model	Variation (R2)	p.value	p.adjusted
Group	9	0.751449232	0.083494359	1.113982187	0.333907054	0.313
S1 vs S2	1	0.030147791	0.777853614	0.162803986	0.7	1
S1 vs S3	1	0.027799065	0.40726882	0.092408436	0.8	1
S1 vs S4	1	0.075249371	1.503679319	0.273213469	0.3	0.794117647
S1 vs S5	1	0.112744474	1.281924962	0.242700336	0.3	0.794117647
S1 vs S6	1	0.024325045	0.383565039	0.087500707	1	1
S1 vs S7	1	0.041082041	0.385741516	0.087953545	0.9	1
S1 vs S8	1	0.024543135	0.576593851	0.125987551	0.8	1
S1 vs S9	1	0.123427038	1.61477758	0.28759422	0.2	0.794117647
S1 vs S10	1	0.045436735	0.874046613	0.179326683	0.5	1
S2 vs S3	1	0.03394918	0.626072677	0.135335677	0.8	1
S2 vs S4	1	0.037154453	1.031729098	0.205044643	0.6	1
S2 vs S5	1	0.100122127	1.354508362	0.252965963	0.3	0.794117647
S2 vs S6	1	0.028013384	0.567225975	0.124194857	0.9	1
S2 vs S7	1	0.043448804	0.469870155	0.105119419	1	1
S2 vs S8	1	0.00929487	0.325746516	0.075304116	0.9	1
S2 vs S9	1	0.182088876	2.917891269	0.42178912	0.1	0.75
S2 vs S10	1	0.08425131	2.219904612	0.356903321	0.1	0.75
S3 vs S4	1	0.053982469	0.824015754	0.170815312	0.5	1
S3 vs S5	1	0.074583321	0.721187972	0.152755615	0.6	1
S3 vs S6	1	0.048107579	0.609834689	0.132289926	0.7	1
S3 vs S7	1	0.03970493	0.325531811	0.075258217	1	1
S3 vs S8	1	0.038439114	0.662358704	0.142065153	0.8	1
S3 vs S9	1	0.121501014	1.322044258	0.248409106	0.3	0.794117647
S3 vs S10	1	0.064429898	0.955192092	0.192765906	0.6	1
S4 vs S5	1	0.052934743	0.621274141	0.134437846	0.7	1
S4 vs S6	1	0.089353178	1.472713713	0.269101179	0.2	0.794117647
S4 vs S7	1	0.026916495	0.259421989	0.060905444	1	1
S4 vs S8	1	0.024698824	0.62026351	0.134248514	0.8	1
S4 vs S9	1	0.262965687	3.568534767	0.471496119	0.1	0.75
S4 vs S10	1	0.132010977	2.681051851	0.401291879	0.2	0.794117647
S5 vs S6	1	0.123903236	1.256901376	0.239095484	0.3	0.794117647
S5 vs S7	1	0.021949467	0.154943066	0.037291261	0.8	1
S5 vs S8	1	0.087071188	1.120236099	0.218786024	0.4	0.9
S5 vs S9	1	0.301053887	2.697713149	0.402781231	0.1	0.75
S5 vs S10	1	0.17758922	2.037873926	0.33751515	0.2	0.794117647
S6 vs S7	1	0.050719861	0.433020287	0.097680646	1	1
S6 vs S8	1	0.02814791	0.529148789	0.116831841	1	1
S6 vs S9	1	0.142092198	1.632026013	0.289776008	0.3	0.794117647
S6 vs S10	1	0.070902291	1.132383661	0.220635037	0.4	0.9
S7 vs S8	1	0.031589067	0.328103094	0.075807597	1	1

pairs	Df	SumsOfSqs	F.Model	Variation (R2)	p.value	p.adjusted
S7 vs S9	1	0.208004923	1.598217372	0.285486837	0.1	0.75
S7 vs S10	1	0.108737758	1.028773677	0.204577446	0.4	0.9
S8 vs S9	1	0.198172959	2.992991796	0.427998757	0.1	0.75
S8 vs S10	1	0.100405816	2.404311764	0.375420787	0.2	0.794117647
S9 vs S10	1	0.054200452	0.716643878	0.151939366	0.6	1
