# Supplementary material for: Diversity and Functional Distribution Characteristics of Myxobacterial Communities in the Rhizosphere of Tamarix chinensis Lour in Ebinur Lake Wetland, China
Source: Microorganisms. 2023 Jul 28;11(8):1924. doi: 10.3390/microorganisms11081924 (PMC10459050; doi:10.3390/microorganisms11081924)
Supplement: Supplementary file 1 [file microorganisms-11-01924-s001.zip › supplementary files/Table S3.pdf]

Table S3

Table S3 Redundancy analysis of myxobacteria genera and soil physicochemical factors

Name	Explains %	Contribution %	pseudo-F	P
EC	9.2	24.8	2.8	0.006
MI	7.7	20.7	2.5	0.02
AK	4.6	12.4	1.5	0.148
pH	4.1	11.1	1.4	0.22
IN	3.7	9.8	1.2	0.26
MC	3.2	8.5	1.1	0.4
CI	2.3	6.2	0.8	0.63
AP	1.7	4.5	0.6	0.836
OM	0.7	1.8	0.2	0.992
