# Supplementary material for: Diversity and Functional Distribution Characteristics of Myxobacterial Communities in the Rhizosphere of Tamarix chinensis Lour in Ebinur Lake Wetland, China
Source: Microorganisms. 2023 Jul 28;11(8):1924. doi: 10.3390/microorganisms11081924 (PMC10459050; doi:10.3390/microorganisms11081924)
Supplement: Supplementary file 1 [file microorganisms-11-01924-s001.zip › supplementary files/Table S4.pdf]

Table S4

Table S4 Redundancy analysis of myxobacterial diversity index and soil physicochemical factors

Name	Explains %	Contribution %	pseudo-F	P
CI	15.6	35	5.2	0.018
MC	11.1	24.9	4.1	0.046
MI	4.3	9.8	1.6	0.168
AK	4.1	9.1	1.6	0.238
pH	2.1	4.7	0.8	0.38
AP	2.7	6.2	1.1	0.348
EC	2.6	5.8	1	0.36
OM	1	2.3	0.4	0.568
IN	1	2.2	0.4	0.622
