# Supplementary material for: Diversity and Functional Distribution Characteristics of Myxobacterial Communities in the Rhizosphere of Tamarix chinensis Lour in Ebinur Lake Wetland, China
Source: Microorganisms. 2023 Jul 28;11(8):1924. doi: 10.3390/microorganisms11081924 (PMC10459050; doi:10.3390/microorganisms11081924)
Supplement: Supplementary file 1 [file microorganisms-11-01924-s001.zip › supplementary files/Table S5.pdf]

Table S5

Table S5 Redundancy analysis of myxobacterial functionand soil physicochemical factors

Name	Explains %	Contribution %	pseudo-F	P
AK	14.3	31.6	4.7	0.046
AP	11	24.3	4	0.036
MI	7.8	17.3	3	0.082
pH	3.5	7.7	1.4	0.242
IN	3.1	6.9	1.2	0.302
OM	2.7	6	1.1	0.336
MC	1.9	4.2	0.7	0.5
EC	0.7	1.6	0.3	0.79
CI	0.2	0.5	<0.1	0.964
